# Bovine leukemia virus (BLV) and risk of breast cancer; a systematic review and meta-analysis

**DOI:** 10.1186/s12977-024-00653-y

**Published:** 2024-12-03

**Authors:** Fateme Saeedi-Moghaddam, Mahdi Mohammaditabar, Sayed-Hamidreza Mozhgani

**Affiliations:** 1 Student Research Committee, Alborz University of Medical Sciences, Karaj, Iran; 2 Department of Microbiology and Virology, School of Medicine, Alborz University of Medical Sciences, Karaj, Iran; 3 Non-communicable Disease Research Center, Alborz University of Medical Sciences, Karaj, Iran

**Keywords:** Breast cancer, Bovine leukemia virus, BLV

## Abstract

**Background:**

The role of viruses in the development of breast cancer has been a subject of debate and extensive research over the past few decades. Several studies have examined the association between Bovine leukemia virus (BLV) infection and the risk of developing breast cancer; however, their findings have yielded inconsistent results. To address this uncertainty, the purpose of the present study was to conduct a systematic review and meta-analysis to determine any potential association between BLV and breast cancer.

**Methods:**

The literature search was performed by finding related articles from PubMed, Web of Science, Scopus, EMBASE, and ScienceDirect databases. Statistical analysis was conducted using the meta package in R Studio and Review Manager 5.1. The I^2^ test was used to assess between-study heterogeneity. The Mantel-Haenszel method calculated the pooled odds ratio and its 95% confidence interval. Studies were divided into subgroups for comparison.

**Results:**

The literature search identified a total of 17 studies that were deemed suitable for inclusion in the systematic review. Out of these 17 studies, 12 were used in the subsequent meta-analysis. Combining the data from these eligible studies, we calculated the pooled multi-factor adjusted odds ratio (OR) and a 95% confidence interval (CI). Considering the heterogeneity observed across the studies, the result obtained using the fixed effects model was 2.12 (1.77, 2.54). However, upon removing the six studies that contributed significantly to the heterogeneity, the pooled OR with a 95% CI was recalculated to be 3.92 (2.98, 5.16).

**Conclusion:**

The result of this study suggests that BLV infection is statistically associated with Breast cancer.

## Introduction

Cancer poses a significant threat to public health and is a leading cause of death worldwide, accounting for nearly 10 million deaths in 2020, which is approximately one in six deaths [1]. Breast cancer is the most frequently diagnosed cancer among women. It holds the position of the 5th leading cause of cancer-related deaths, with an estimated 2.3 million new cases reported worldwide, according to the GLOBOCAN 2020 data [2].

Epidemiologic studies have established a wide range of risk factors for breast cancer. Among them are well-known factors such as female gender, race, ethnicity, family history, and genetic mutations. In addition, certain modifiable factors can also contribute to the risk, including increased alcohol intake, physical inactivity, high body mass index (BMI) and use of exogenous hormones [3].

Although the risk factors for breast cancer are well established, the exact causes of breast cancer are still unknown. However, in recent years, there has been growing evidence suggesting that certain viruses may have an influential role in the development of breast cancer. Notably, Human Papilloma Viruses (HPVs), Epstein–Barr virus (EBV), Mouse Mammary Tumor Virus (MMTV), and Bovine Leukemia Virus (BLV) have emerged as potential oncogenic viruses that could contribute to the pathogenesis of breast cancer [4–7].

BLV is a delta retrovirus closely related to the human T-cell leukemia virus 1. BLV contains the typical retroviral genome regions, including *LTR*, *gag*, *pol*, and *env*. However, unlike other oncogenic retroviruses, delta retroviruses have an additional region called tax, which has regulatory functions and is oncogenic to host cells. Tax induces malignant transformation by inhibiting DNA repair and disrupting cellular growth control mechanisms [8].

While BLV is primarily found in cattle and is considered a zoonotic virus, some evidence also suggests its presence in humans. BLV has been detected and identified in breast cancer samples through various methods such as RT-PCR, In-situ PCR assay, ELISA, immunohistochemistry, in situ hybridisation, and DNA sequencing [7, 9]. The presence of the virus in women’s breast tissue and blood suggests transmission from cattle to humans [10]. Furthermore, antibodies against BLV have been isolated in humans, providing additional evidence of its possible transmission [11].

It is worth noting that although there are pathways that suggest BLV can contribute to the development of breast cancer, there is currently no conclusive evidence to support this. The findings of different studies have been conflicting, and the presence of BLV in breast cancer cases can vary across different regions.

Due to the ongoing controversy surrounding the association between BLV and breast cancer, there is debate about the virus’s causal role in the development of this type of cancer. To shed light on this issue, the present study was conducted to explore any possible relationship between BLV and breast cancer. We aimed to provide clarity by conducting a systematic review and meta-analysis, examining the existing evidence on this topic.

## Methods

### Search strategy

This study was performed under the PRISMA guidelines [12,13]. All relevant studies were identified by exploring online databases including MEDLINE (PubMed), Web of Science, Scopus, EMBASE, and ScienceDirect from 2005 to October 2024. The following keywords were used to find the related reports: “Bovine leukaemia virus”, “Bovine leukemia virus”, “BLV”, “Breast cancer”, “Breast carcinoma”, “Breast gland cancer”, “breast gland neoplasm”, “mammary cancer” and “mammary gland cancer”.

Two independent reviewers screened and evaluated the studies. The agreement between authors to evaluate and select the articles was surveyed by calculating the kappa coefficient.

### Inclusion and exclusion criteria

The inclusion criteria were studies that assessed the prevalence of BLV in breast cancer-diagnosed patients and case-control studies that discussed their association. The exclusion criteria were reviews, studies with possible duplicate samples and full text of non-English articles. Non-English articles that had an English abstract were included.

### Data extraction and quality assessment

Two independent reviewers extracted data from the included studies by developing a data extraction sheet according to the rationale suggested by the Cochrane Consumers and Communication Group Data Extraction Template (available at http://cccrg.cochrane.org/author-resources). The duplicate studies identified from various databases were considered only once. The details of each study including authors, year of publication, sample collection date, country, continent, study design, sample type, detection target, total cases, positive cases, total controls, positive controls, detection method, age of cases and controls and finally NOS score were collected and considered at 3 levels: title, abstract, and full text.

To evaluate the quality and risk of bias in case-control studies, the Newcastle-Ottawa quality assessment scale (NOS) was used. The Cochrane Collaboration recommends NOS, which consists of eight items. These items are divided into three dimensions: selection, comparability, and outcome or exposure for case-control studies [14]. A score ≥ 6 was considered as the high-quality score (low risk of bias), scores of 4–6 as the moderate-quality score (moderate risk of bias), and scores < 4 as the low-quality score (high risk of bias). Meetings were held to calibrate everyone involved in the study, discuss research instruments, and interpret NOS items. Two researchers independently analysed each study, with divergences resolved by a third researcher.

### Assessment of heterogeneity and statistical analysis

Meta-analysis and statistical analysis were performed using a meta package in R studio and Review Manger 5.1. Between-study heterogeneity was investigated using the *I*^2^ test since an *I*^2^ of around 25%, 50%, and 75% is considered low, moderate, and high levels of heterogeneity, respectively. Using the Mantel-Haenszel method, the pooled odds ratio and 95% confidence interval were calculated. To explore the patterns of heterogeneity, Graphic Display of Heterogeneity (GOSH) plots illustrated by metafor package in R studio and DBSCAN, K-Mean, and Gaussian Mixture Model, unsupervised learning algorithms, were used to find outlier studies. Then a new forest plot without outlier studies was drawn. The results of these two forest plots were compared. Lastly, studies were divided into subgroups for further methodological comparison.

## Results

### Literature search and study characteristics

**Fig. 1:**
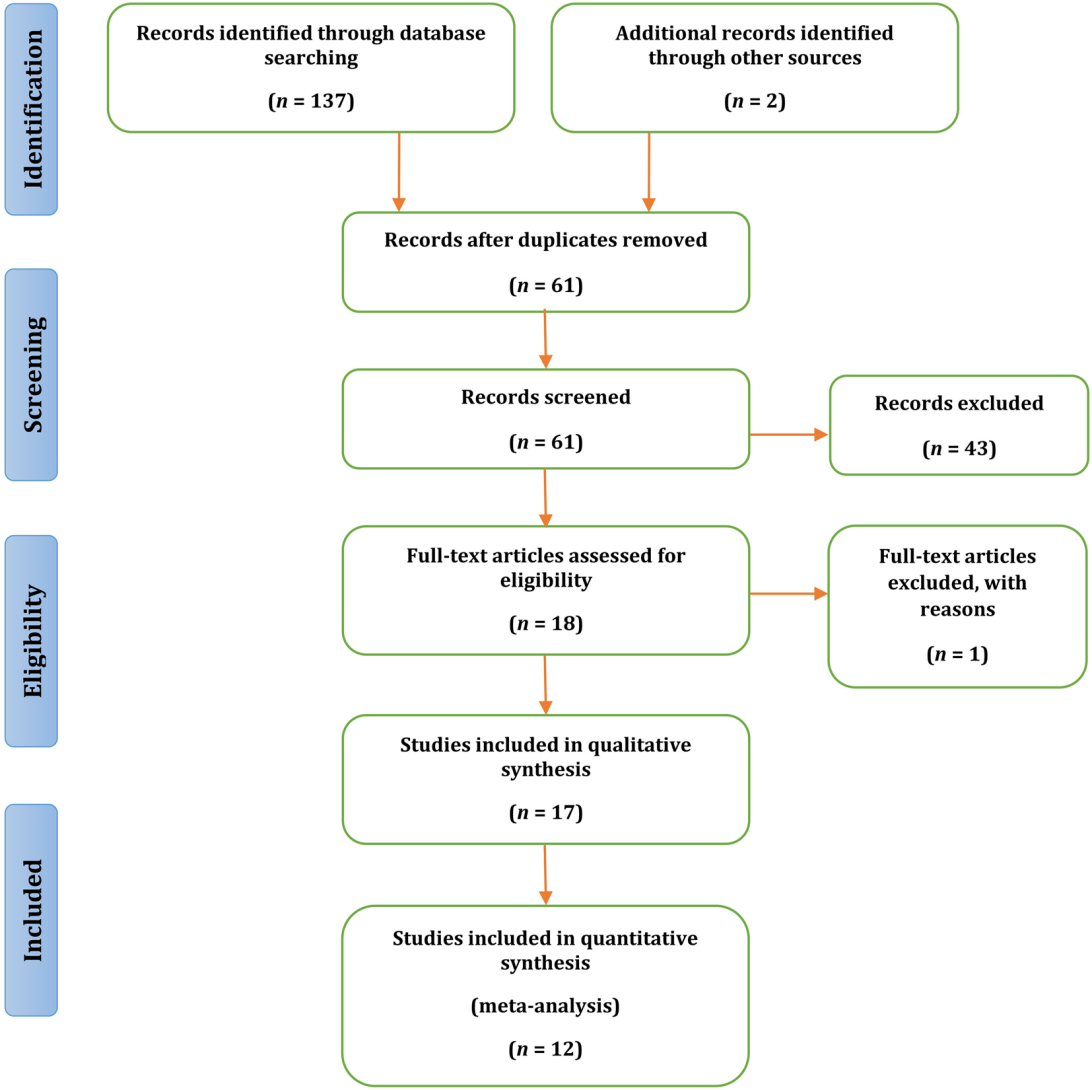
Schematic flowchart of the conducted steps for selecting studies based on the PRISMA statements

The detailed steps of the search and study selection are presented in Fig. 1. The primary search identified a total of 139 records in MEDLINE (46), Scopus (42), Web of Science (30), EMBASE (9), ScienceDirect (10) and grey literature search (2) based on the title screening. After removing duplicate reports, 61 studies were retained. Further screening of the titles and abstracts led to the exclusion of 43 studies and the inclusion of 18 studies. Following a full-text evaluation, 1 additional study was excluded. As a result, 17 studies were deemed eligible for qualitative synthesis, and 12 were selected for the meta-analysis. The characteristics of the selected studies are shown in Table 1. The kappa coefficient of 0.95 revealed the perfect agreement between 2 investigators.

**Table 1:** Characteristics of the studies included in the meta-analysis

study	Publication year	Sample collection date	country	continent	Study- design	Sample type	Detection target	Case	Case positive	Control	Control positive	Detection method	Age control	Age case	NOS score
Buehring [15]	2015	2002–2008	US	North America	Case– control	FFPE	tax	114	67	104	30	In-situ PCR	48.7	55.9	9
Baltzell [16]	2017	2009–2011	US	North America	Case– control	FFPE	tax	61	35	103	20	In-situ PCR	54.4	54.59	9
Giovanna [17]	2013	2006–2013	Colombia	South America	Case– control	FFPE	gag	53	19	53	24	PCR	52.2	46.4	7
Olaya 1 [18]	2021	2016–2018	Colombia	South America	Case– control	FFPE	gag LTR tax env	75	33	83	30	Nested PCR	40.55	66.15	9
Olaya 2 [18]	2021	2016–2018	Colombia	South America	Case– control	Blood	gag LTR tax env	75	28	83	20	Nested PCR	40.55	66.15	9
Olaya 3 [18]	2021	2016–2018	Colombia	South America	Case– control	FFPE	tax	75	21	83	19	In-situ PCR	40.55	66.15	9
Schwingel [19]	2019	2015–2017	Brazil	South America	Case– control	FFPE	tax	72	22	72	10	Nested PCR	38	52.2	9
Delaramina 1 [20]	2020	2011–2018	Brazil	South America	Case– control	FFPE	tax	49	46	39	23	Nested PCR	NR	NR	9
Delaramina 2 [20]	2020	2011–2018	Brazil	South America	Case– control	FFPE	env	49	28	39	14	Nested PCR	NR	NR	9
Buehring [21]	2017	1995–2010	Australia	Oceania	Case– control	FFPE	tax	50	40	46	19	In-situ PCR	49.59	55.08	9
Lawson and Glenn [22]	2017	NR	Australia	Oceania	Case– control	FFPE	tax	22	20	17	6	In-situ PCR	36.4	56.1	8
Khalilian 1 [10]	2019	2017–2018	Iran	Asia	Case– control	FFPE and Blood	tax	172	52	200	60	In-situ PCR	47.5	53	9
Khalilian 2 [10]	2019	2017–2018	Iran	Asia	Case– control	FFPE and Blood	gag	172	14	200	16	Nested PCR	47.5	53	8
Dabaghi 1 [23]	2023	2022	Iran	Asia	Case– control	FFPE	gag	50	8	50	0	Nested PCR	NR	NR	8
Dabaghi 2 [23]	2023	2022	Iran	Asia	Case– control	Blood	gag	60	11	60	5	Nested PCR	NR	NR	8
Khan [24]	2022	NR	Pakistan	Asia	Case– control	FFPE	tax gag	2710	728	80	10	Nested PCR	NR	NR	8
Khasawneh [25]	2024	2018–2022	Jordan	Asia	Case– control	FFPE	tax	103	19	25	0	Nested PCR	20–80	56	8

### Quality assessment

The quality assessment of the studies according to the Newcastle-Ottawa Scale is summarised in Table 1.

#### Publication bias and heterogeneity

**Fig. 2:**
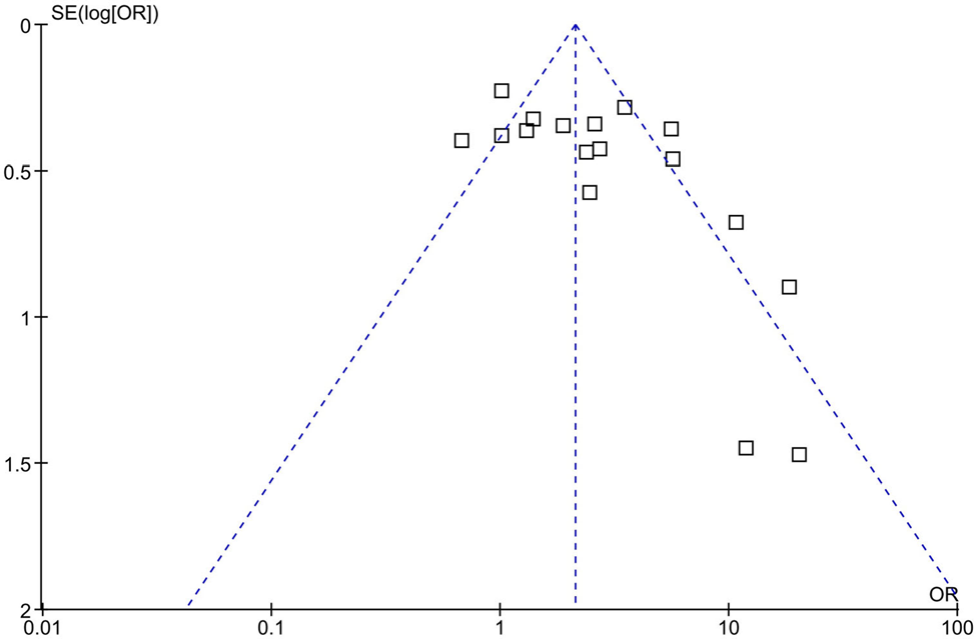
Funnel plot of included studies for investigation of publication bias

**Fig. 3:**
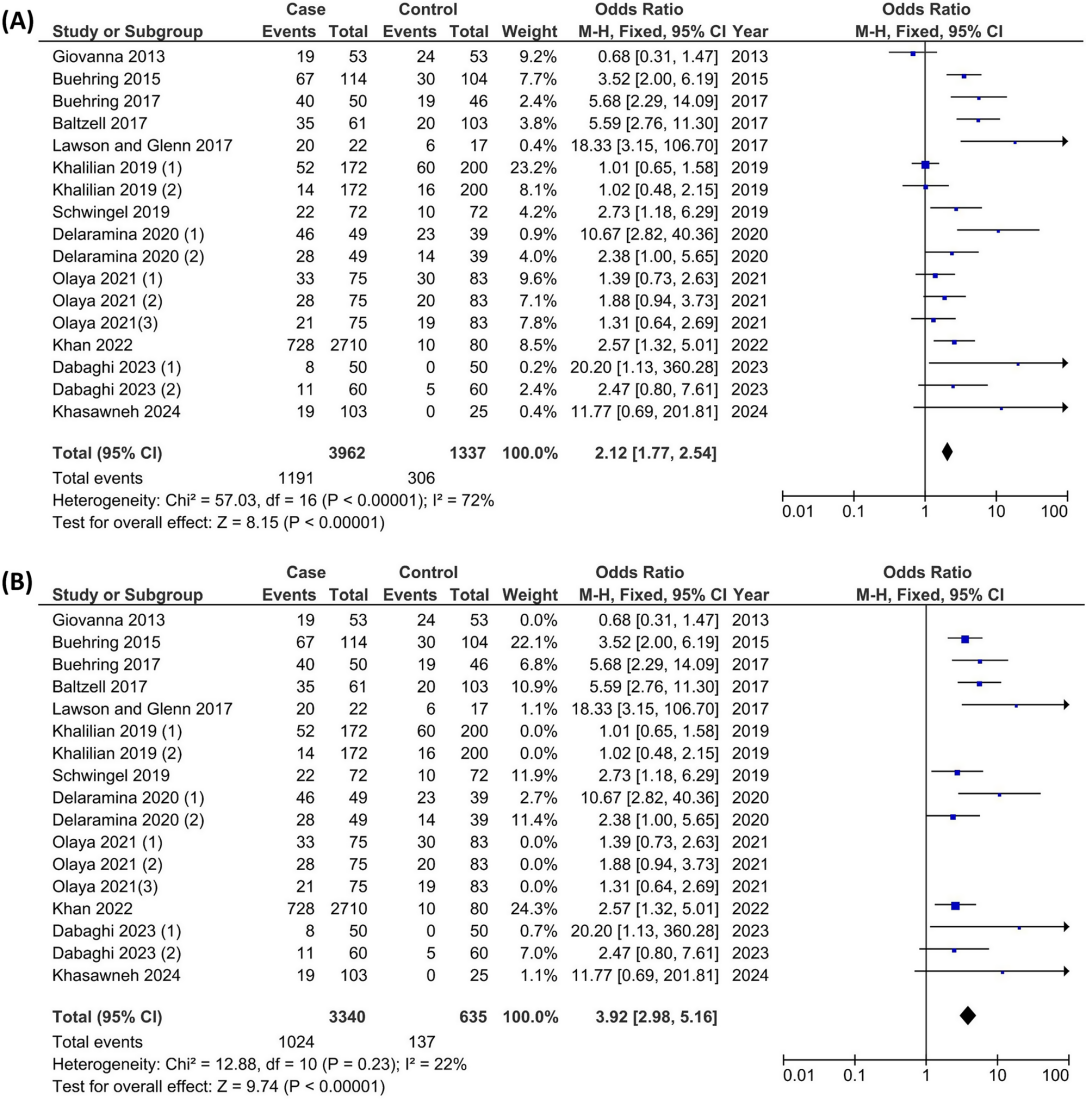
Initial Forest plot of the association between BLV infection and breast cancer risk (**A**). Final forest plot of the association between BLV infection and breast cancer risk after excluding the outlier studies (**B**)

A sensitivity analysis was conducted to mitigate potential publication bias. This involved systematically excluding each study to evaluate its impact on the overall meta-analytic results, including the main summary estimate and the I² statistic for heterogeneity. The findings were consistent across analyses, demonstrating a relatively low sensitivity to these exclusions and reinforcing the credibility of the meta-analysis results. The illustrated Funnel plot showed us that some studies were out of the expected line and identified potential outliers [10, 17, 18] (Fig. 2). The results of the initial forest plot, which included 17 case-control studies [fixed effects model OR = 2.12, 95%-CI [1.77; 2.54], p-value < 0.00001], had high levels of heterogeneity [I^2^ = 72%], which also showed there could be some outlier studies (Fig. 3-A). The identified outliers were excluded and the forest plot was illustrated again, as the results were identical [fixed effects model OR = 3.92, 95%-CI [2.98; 5.16], p-value < 0.00001] although with significantly lower levels of heterogeneity [I^2 = 22%] they validated each other (Fig. 3-B).

**Fig. 4:**
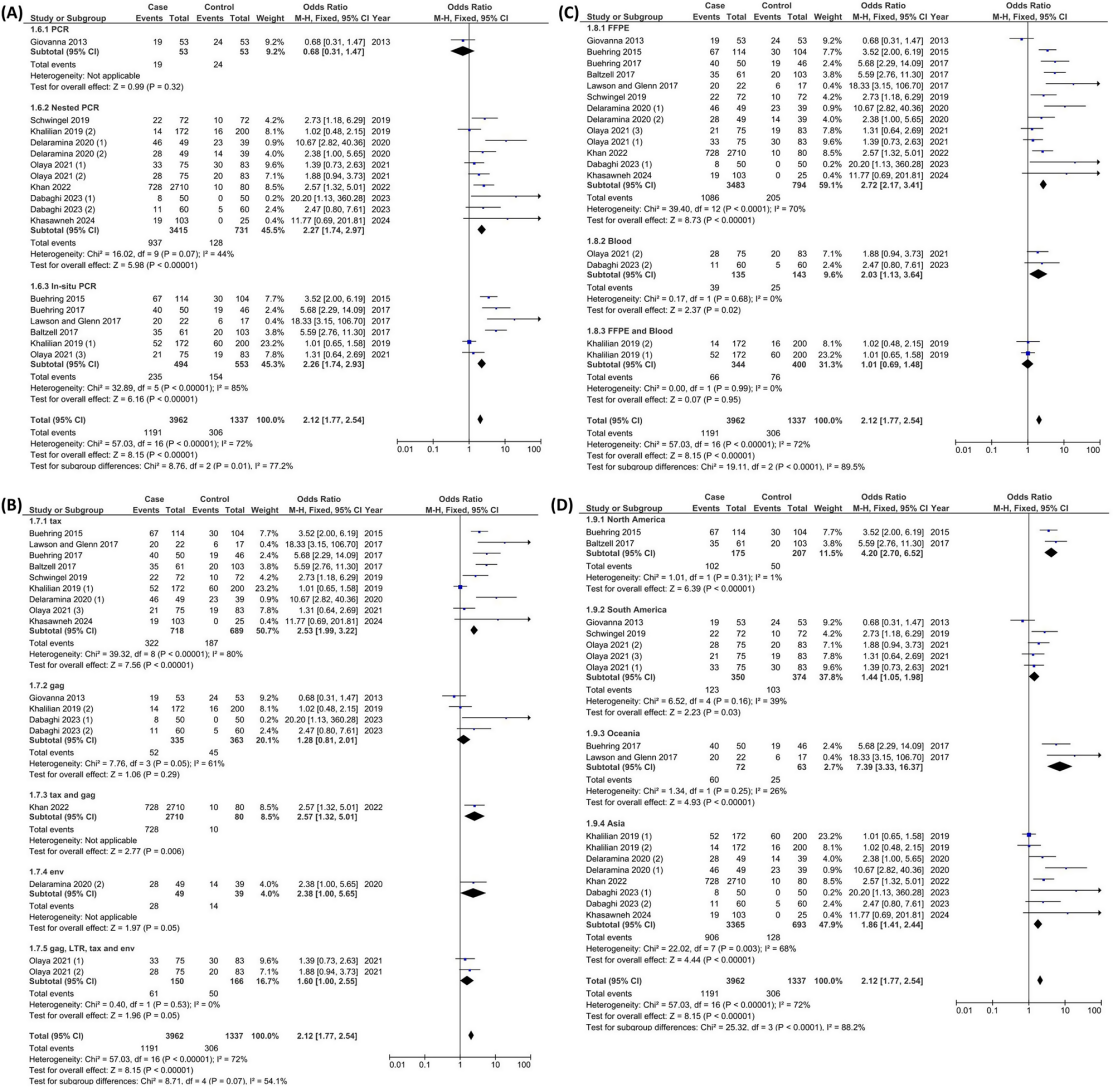
Forest plot of the association between BLV infection and risk of breast cancer based on subgroups: detection method (**A**), detection target (**B**), sample type (**C**), and study location (**D**)

The possible parameters associated with the detection rate of BLV were examined in the subgroup analysis (Fig. 4). These parameters include detection method, detection target, sample type, and study location. Although our initial forest plot and funnel plot revealed outliers, these were not excluded from the subgroup analysis to avoid selection bias. The subgroup analysis of the forest plots indicated that the PCR method and the gag gene as the target were not the best choices for these studies, as both demonstrated controversial results compared to others (Fig. 4-A, B). Khalilian et al. reported results that differed from those of other sample type subgroups, likely due to their investigation of BLV in formalin-fixed, paraffin-embedded (FFPE) tissues for cases and blood samples for controls [10] (Fig. 4-C). The interpretation of results would have been more straightforward if the same sample types had been used for both cases and controls. Furthermore, subgroup analysis based on study location did not produce conflicting results (Fig. 4-D). This may be attributed to the exclusion of studies with insufficient data—those with the most contentious outcomes—from the search strategy, preventing their inclusion in the meta-analysis.

### Global research on BLV’s role in breast cancer

To explore the relationship between BLV and breast cancer, several case-control studies have been conducted across different countries. Summarising the results from these studies can help us better understand regional and ethnic factors that may influence the connection between BLV infection and breast cancer development.

#### USA

In 2007, Buehring and co-authors published the first research paper reporting the presence of BLV DNA and proteins. The results showed that BLV was detected in 59% of breast cancer cases and 29% of controls. Interestingly, among the breast cancer samples, 69% exhibited BLV proviral DNA in accompanying non-malignant mammary epithelium. This finding suggested that the development of cancer might have been a rare and delayed event within a population of BLV-infected cells in breast tissue. These results provided an encouraging initial step in establishing a causal link between BLV and human breast cancer [26].

In a similar study conducted in 2015, Buehring et al. performed research to investigate the presence of BLV DNA in breast tissue samples. The results demonstrated that BLV DNA was detected in 59% of mammary epithelium samples from American women with breast cancer, which was significantly higher than the 29% found in normal controls. Notably, the frequency of BLV DNA in samples from women with premalignant breast cancer was found to be 38%, falling between the frequencies observed in breast cancer and normal-control samples. These findings support the hypothesis that BLV plays a role in developing cancer [15].

According to a study by Baltzell et al., women diagnosed with breast cancer in Texas were significantly more likely to have BLV DNA in their breast tissue compared to women with benign diagnoses or no history of breast cancer. Women with premalignant breast pathology but no cancer history were found to have an increased risk of having BLV DNA in their breast tissue. The study’s attributable risk of 51.82% suggests that BLV might be responsible for at least half of the breast cancer cases in the studied population. It is worth noting that Texas, where the study subjects are from, is known for its high beef and dairy consumption and thriving cattle industry. 38% of buffy coat cells from the West Coast of the United States have been found to contain BLV. This supports previous findings that have revealed a significant association between BLV DNA in breast tissue and a breast cancer diagnosis [16].

In contrast to previous results, a recent study by Amato et al. found no evidence of BLV DNA in fresh-frozen breast cancer tumours from patients at a hospital in Vermont. This study suggests a low prevalence of BLV in the patient population [27]. However, it is important to note that the reliability and accuracy of these negative results are still to be determined.

#### Colombia

Giovanna et al. conducted a case-control study that interestingly showed a higher percentage of BLV DNA detection in the control group. The researchers noted that while the presence of BLV genes in human breast tissue was confirmed, it should be clarified as a possible promoter of malignancy processes in this tissue. The study authors raised the point that their results seemingly contradicted the findings of previous studies [17].

Olaya-Galán et al. conducted an observational case-control study in which the researchers used Nested PCR, In-situ PCR, and immunohistochemistry to detect BLV in blood and breast tissues. The results showed that BLV was more prevalent in the cases group (61.3%) compared to the controls (48.2%). The study confirmed a statistically significant association between BLV and breast cancer, with the virus found in both the blood and breast tissues of participants. Therefore, BLV was identified as an intermediate risk factor for breast cancer in Colombia [18].

#### Brazil

Schwingel et al. investigated the presence of the BLV genome in breast cancer tissues in south Brazil. The results showed that BLV DNA was more common in breast cancer tissue (30.5%) compared to healthy breast tissue (13.9%). The researchers suggested that the link between BLV and breast cancer is stronger than the links to lifestyle and reproductive history. They also noted that dairy products are more commonly consumed in south Brazil than in comparison to other regions of the country. Based on these findings, the study suggests that BLV may be a potential factor that increases the risk of breast cancer in women [19].

A study conducted by Delaramina et al. amplified BLV proviral genes from breast tumour samples and healthy control samples from women. The results showed a positivity rate of 95.9% in tumour samples and 59% in healthy tissue samples. This evidence confirms the presence of the BLV genome in the breast tissues of women in the state of Minas Gerais. It indicates a statistically significant positive association between BLV infection and breast cancer within this population [20].

#### Australia

In a study by Buehring and colleagues, it was revealed that 80% of women who had been diagnosed with breast cancer had BLV DNA in their breast tissue, compared to only 41% of women with no history of breast cancer. The results also showed that 60.4% of women who tested positive for BLV and had not been diagnosed with breast cancer later developed the disease, whilst only 14.6% of BLV-negative women developed breast cancer. Hence, it suggests a possible temporal relationship between BLV infection and the subsequent development of cancer. In 74.2% of breast cancer patients, BLV infection was present years before the diagnosis, indicating that BLV might play a role in the development or acceleration of breast cancer [21].

A study conducted by Lawson and Glenn found that BLV was present in 78% of 23 benign breast specimens and 91% of 22 subsequent breast cancers in the same patients. The presence of BLV was confirmed by sequencing the products of standard PCR. As the prevalence of this virus is so high in both benign and later breast cancer cells, it is likely to be also present in other virus-positive benign and cancerous cells [22].

#### Iran

Khalilian et al. conducted a study involving 400 samples, comprising 200 breast cancer-suspected tissue samples and 200 blood samples from women without breast cancer, collected from two hospitals in Qom Province, Iran. Of the breast cancer-suspected samples, 172 were confirmed malignant. Using Nested PCR, the study detected BLV tax and gag genes in 30% and 8% of the malignant tissue samples, respectively. Additionally, 16.5% of the blood samples from women without breast cancer tested positive for BLV. The authors proposed that the Nested PCR technique could help establish a connection between human breast cancer and BLV infection in cattle [10].

A recent study conducted by Dabaghi et al. in 2022 investigated the presence of BLV in breast tissue and blood samples. The study used the Nested PCR method to detect BLV infection and showed that 13% of the blood samples and 8% of the breast paraffin tissue samples were infected with BLV. Although there was a notable relationship between BLV infection and breast cancer in the studied population’s paraffin tissue samples, more blood samples tested positive for this virus. Therefore, blood samples are preferable for detecting this virus in patients [23].

#### China

A study conducted in China by Zhang et al. had findings that contradicted the previous dominant beliefs. The researchers stated that there was no association between breast cancer and BLV. However, it is crucial to be cautious in interpreting these findings. The Chinese scientists used a commercial BLV testing kit designed for cows on human blood samples, which could have affected the results. As Buehring and colleagues pointed out, these commercial kits are not intended for testing human sera [28]. Some human sera could yield negative results because the final detection step involves a labelled antibody to bovine rather than human immunoglobulin [29].

#### Japan

A study by Saito et al. reported the absence of BLV DNA in Japanese human cell lines. The authors examined DNA extracted from 145 cell lines but did not detect BLV DNA. This raises questions about the optimality of the protocol as previous research has shown the presence of BLV DNA in some proportion, not 0%. Saito et al. pointed out that a potential flaw was using a PCR method designed for sheep cell lines, not humans. It was mentioned earlier that Buehring et al. raised concerns about the study by Zhang et al., where a bovine ELISA was used on human samples [28, 29]. Additionally, Saito et al. admitted that they only used two breast cancer cell lines [30].

Similarly, Yamanaka et al. used PCR to examine the presence of BLV proviral DNA in human blood and breast cancer tissue samples and found all the samples yielded negative results [31].

#### Pakistan

A recent study by Khan et al. examined the presence of BLV in human breast tissue through Nested PCR by amplifying tax and gag genes. The study results showed that BLV genes were positive in 26.8% of the samples from breast cancer patients, while only 10% of the samples without cancer were positive. Therefore, the study suggests that there may be a relationship between the presence of the BLV gene and breast cancer [24].

#### Jordan

The results of a study by Khasawneh et al. showed that BLV was detected in 18.4% of the breast cancer samples and none of the control samples tested positive for BLV. The study also investigated the relationship between BLV and breast cancer molecular subtypes, finding the most positive cases in luminal A and luminal B patients. However, the correlation with the HER2 subtype was not statistically significant due to a small sample size. It was observed that larger tumours were more frequently associated with metastatic tissue in sentinel lymph nodes. Most patients had tumours smaller than 5 cm, with a notable prevalence of grades 2 and 3, which are indicative of a poorer prognosis. However, no significant correlation with BLV DNA was identified, likely due to the small sample size analysed [25 ].

## Discussion

The relationship between BLV and breast cancer has been a topic of debate over the last few decades. The conflicting results in various studies may be attributed to discrepancies in the methodologies or techniques used to detect BLV in breast samples. It is important to note that different assays have varying sensitivities and standards for diagnosing a sample as ‘BLV positive’.

The most commonly utilised techniques for detecting BLV in breast tissues and blood are polymerase chain reaction (PCR), Nested PCR, and In-situ PCR. Additionally, contradictory outcomes might arise from using different detection targets, such as tax, gag, LTR, and env.

It is crucial to investigate the potential connection between breast cancer and BLV infection. This relationship can not only help us understand the causes of breast cancer better, but it can also aid in early detection, prevention and treatment. Our study aimed to achieve this by pooling data published from 2005 to 2024, presenting a systematic review and meta-analysis of studies investigating the contributions of BLV to the development of breast cancer. Our results show that BLV is associated with increased risks of breast cancer. These findings are consistent with a recent systematic review and meta-analysis examining the relationship between BLV and breast cancer [7]. In this study, we evaluated several potentially important parameters that could impact the detection of Bovine BLV in breast cancer tissues. These parameters include the DNA detection method, the detection of specific regions or genes in the BLV genome, the type of sample used, and the geographic location of the study. Additionally, our research addresses the conflicting global findings concerning the role of BLV in breast cancer.

Though our study found a positive correlation between BLV and breast cancer, the presence and implication of BLV infection in the initiation and progression of breast cancer remains controversial. As mentioned earlier, the conflicting results may be explained by using different technical approaches for detecting BLV.

Several studies have reported a positive correlation between BLV and breast cancer. However, in contrast to these findings, Zhang et al. [29] did not detect BLV using PCR in Chinese breast cancers. It is important to consider that the methods employed in this study may not have been adequate [28], potentially affecting its ability to identify BLV presence in the samples accurately.

Two studies used whole-genome sequencing method and did not identify BLV in breast cancer samples [32, 33]. The exact reason behind these negative results from whole-genome sequencing remains unclear. However, one plausible explanation could be that whole-genome sequencing techniques may not be as sensitive as amplification techniques like PCR [34].

Based on our findings, it appears that relying solely on PCR for BLV detection may not be the most optimal approach. Previous studies that employed PCR to identify BLV in blood or breast tissue either observed no presence of the virus [29, 31] or detected higher levels of the virus in the control group rather than the cases, suggesting a negative correlation between breast cancer and BLV [17]. It is important to acknowledge that PCR analyses are prone to contamination, potentially leading to false positive outcomes. Moreover, the issue of false negative results arises when PCR methods are employed to identify low concentrations of retroviruses. [34]. To address these challenges, several alternative approaches can be considered. Implementing In-situ PCR or Nested PCR methods may yield more accurate results, surpassing the limitations associated with conventional PCR techniques. These alternative methods can mitigate contamination issues and enhance detection sensitivity, providing a more reliable means of identifying BLV in the context of breast cancer research.

Furthermore, the racial and geographic diversity across different studies could contribute to the inconsistencies observed. Factors such as genetic variations and diverse environmental exposures in different populations may influence the association between BLV infection and breast cancer.

BLV and BLV-infected cells are commonly found in colostrum and milk obtained from infected cows [8], thus serving as the primary mode of transmission from cows to humans. Several studies have shown a strong correlation between breast cancer mortality rates and the consumption of bovine meat and milk [35]. Moreover, studies have shown that women with lactose intolerance and lower intake of milk and dairy products have a reduced risk of developing breast cancer compared to those who consume more [36]. While this may suggest the involvement of a virus transmitted through milk or dairy products, other factors such as caloric restriction, the presence of growth factors in milk fats, alterations to the gut microbiome, or the protective effects of dietary factors like plant milk (such as soy and rice milk) should also be considered [37–40].

Countries with higher levels of bovine meat and milk consumption, such as the US, UK, Australia, and Germany, exhibit higher breast cancer rates compared to countries with lower consumption, such as India, Japan, Korea, and China [35].

In the case of India, where the consumption of beef is prohibited, milk consumption has increased over the years, coinciding with an increase in the incidence of breast cancer [35].

Similarly, Japan and Korea have experienced a notable increase in breast cancer rates in recent years, and this could be attributed to the increased consumption of dairy products in these countries, as well as the popularity of raw meat consumption [35]. However, it is essential to consider other potential factors that may contribute to the rise in breast cancer incidence in these regions before solely attributing it to milk and meat consumption.

In China, the rates of breast cancer have historically been relatively low. However, in recent years, there has been an increase in breast cancer cases [35]. One contributing factor to this trend could be the dietary habits in China. A study by Yongfa et al. indicated that 92.3% of Han Chinese individuals (the ethnic majority in China) have lactose malabsorption [41], which has led to a lower popularity of dairy products in comparison to other countries. It is worth noting that milk consumption in China is only a fraction of what it is in countries like Argentina, Australia, New Zealand, and the United States [42]. These findings highlight the intriguing relationship between lactose intolerance, milk consumption, and breast cancer rates in China. Further studies can help to explore the underlying mechanisms and provide insights into potential preventative strategies.

Supporting evidence comes from the Swedish Cancer Registry data and a study conducted by Ji et al. This research indicates that individuals with lactose intolerance tend to exhibit lower incidences of breast cancer compared to their genetically related family members who do not have lactose intolerance and share similar environmental factors [43].

The contradiction between the results of studies could be attributed to genetic variations and differences in lifestyle and dietary preferences among different populations. In the study conducted by Buehring et al., it was observed that women of African descent had lower frequencies of BLV compared to other populations [15].

In a study by Khalilian et al., the significance of maintaining proper hygiene when consuming milk and dairy products was emphasised. The study found that a majority of individuals testing positive for BLV were from regions with relatively poor hygiene practices. In these areas, the consumption of unpasteurised raw milk and dairy products is prevalent due to their lower cost compared to pasteurised alternatives. Regrettably, this increases the risk of BLV transmission from cattle to humans [10]. Additionally, the researchers made an interesting observation that BLV DNA was detected in certain breast cancer samples even after chemotherapy treatment had been administered [10].

It has been suggested that BLV-infected blood cells could potentially spread the virus to different organs, leading to the later formation of cancer [16]. A study conducted by Baltzell et al. revealed that BLV can infect various cell types, including platelets, leukocytes, CD5 + B lymphocytes, T cells, and mammary epithelial cells [16]. Moreover, Robinson LA et al. detected BLV in 80% of squamous cell lung carcinomas. Although squamous cell lung carcinomas differ biologically from breast cancer, this finding suggests a potential link between the two [44, 45].

Based on our study, there is a statistically significant association between BLV infection and an increased risk of breast carcinoma. BLV infection might play a role in breast cancer oncogenesis, although it is not yet known if BLV acts as a primary cause. Discrepancies among studies may be attributed to differences in sample types (FFPE or blood), study populations (Asian, European, American, etc.), and detection methods and targets for BLV. Considering these factors and striving for standardised methodologies, future studies can help clarify the relationship between BLV infection and breast carcinoma. It is important to note that language barriers and limitations in female-focused studies were significant limitations of this study.

The potential connection between BLV and human breast cancer holds great significance due to the prevalent consumption of beef, cow’s milk, and dairy products throughout various regions, including Western countries and increasingly in Asian countries such as Japan and China. Since these food items have become almost universally consumed over a lifetime, it is imperative to conduct further research to investigate the potential role of BLV in breast cancer development. This exploration is crucial for advancing our understanding of the potential public health implications associated with BLV and its potential impact on breast cancer.

## Data Availability

No datasets were generated or analysed during the current study.
